# Supplementary material for: Modulation of Epithelial–Mesenchymal Transition Is a Possible Underlying Mechanism for Inducing Chemoresistance in MIA PaCa-2 Cells against Gemcitabine and Paclitaxel
Source: Biomedicines. 2024 May 3;12(5):1011. doi: 10.3390/biomedicines12051011 (PMC11118094; doi:10.3390/biomedicines12051011)
Supplement: Supplementary file 1 [file biomedicines-12-01011-s001.zip › biomedicines-2957862-supplementary.pdf]

Fig. S1 M-A plot and volcano plot for MIA capa-2-PA vs -GR.

Fig S1. PA vs GR

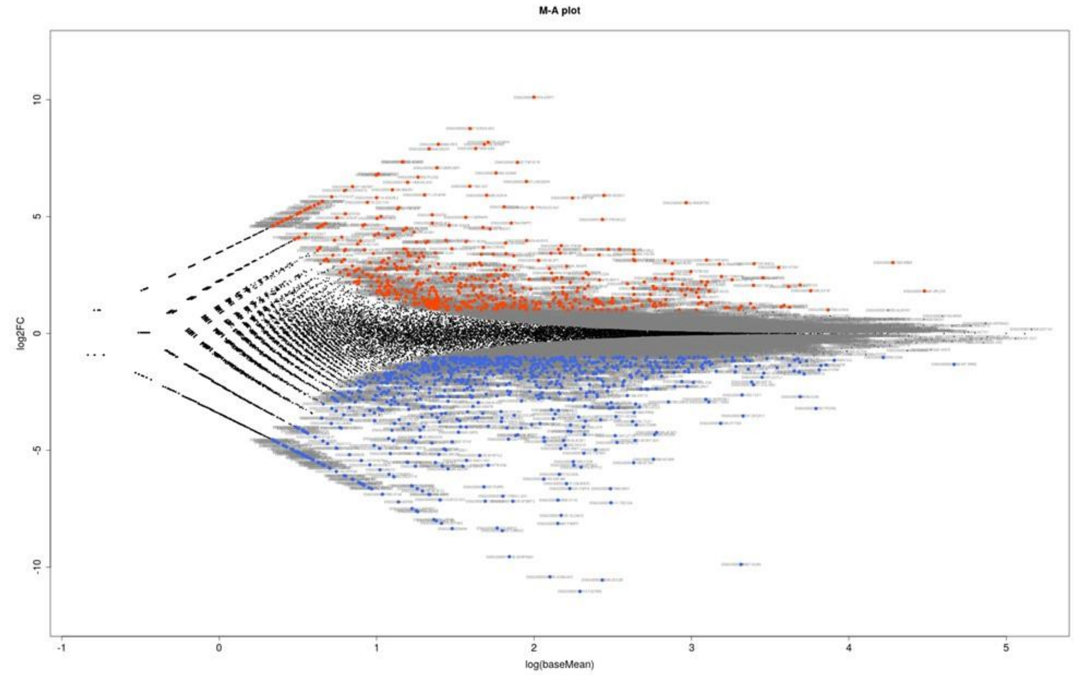

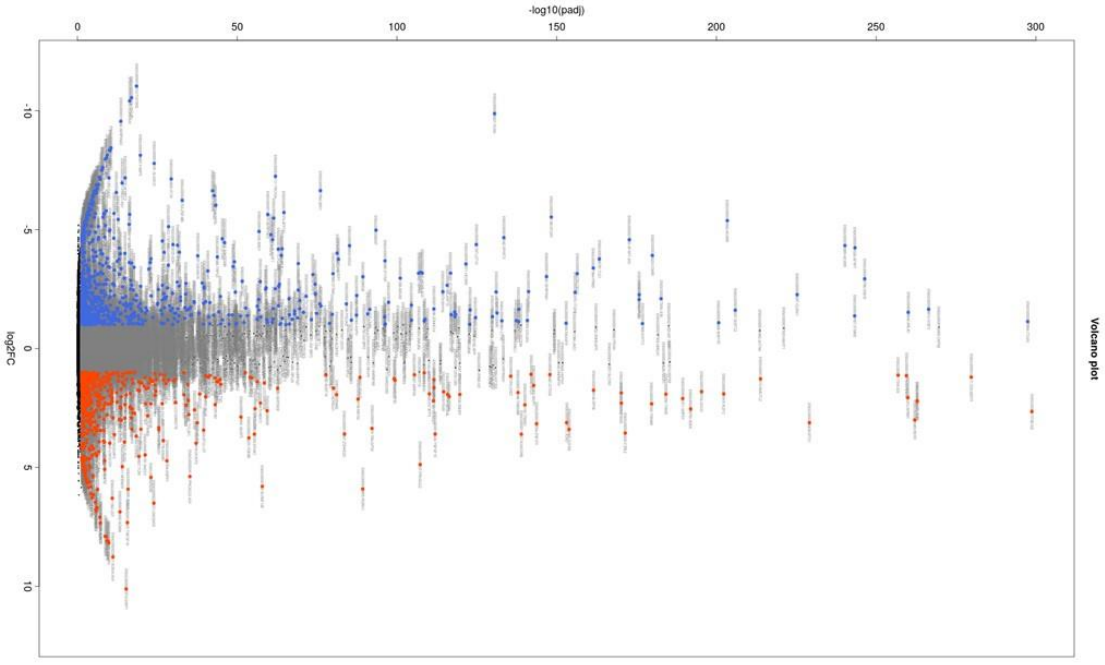

Fig. S2 M-A plot and volcano plot for MIA capa-2-PA vs -PR

Fig. S2. PA vs PR

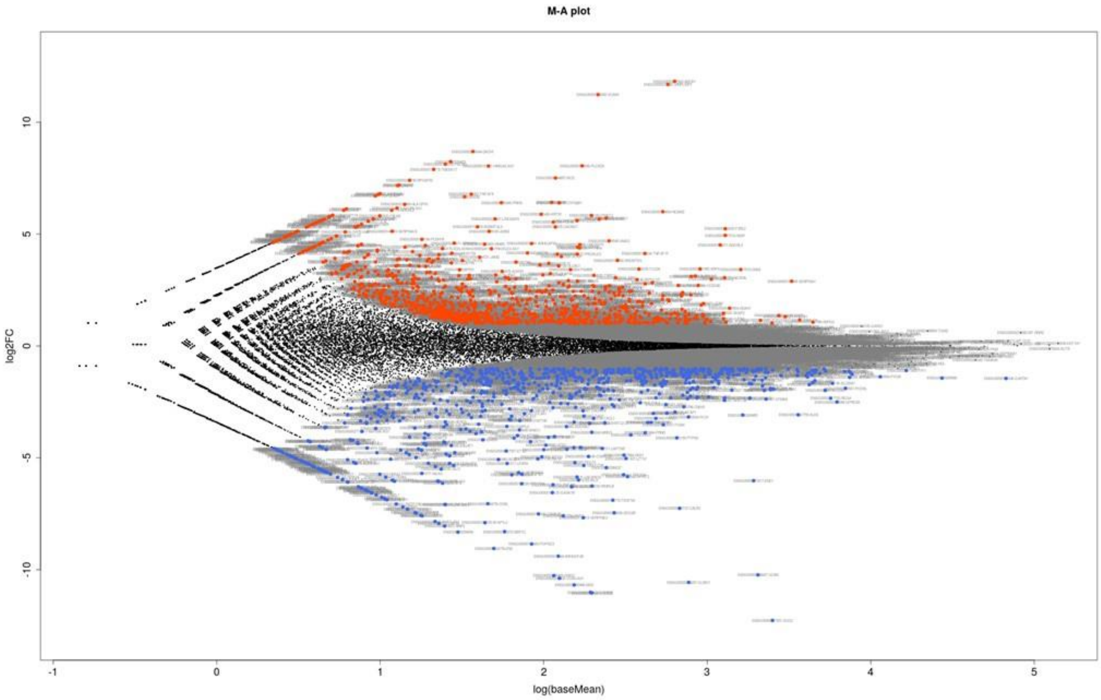

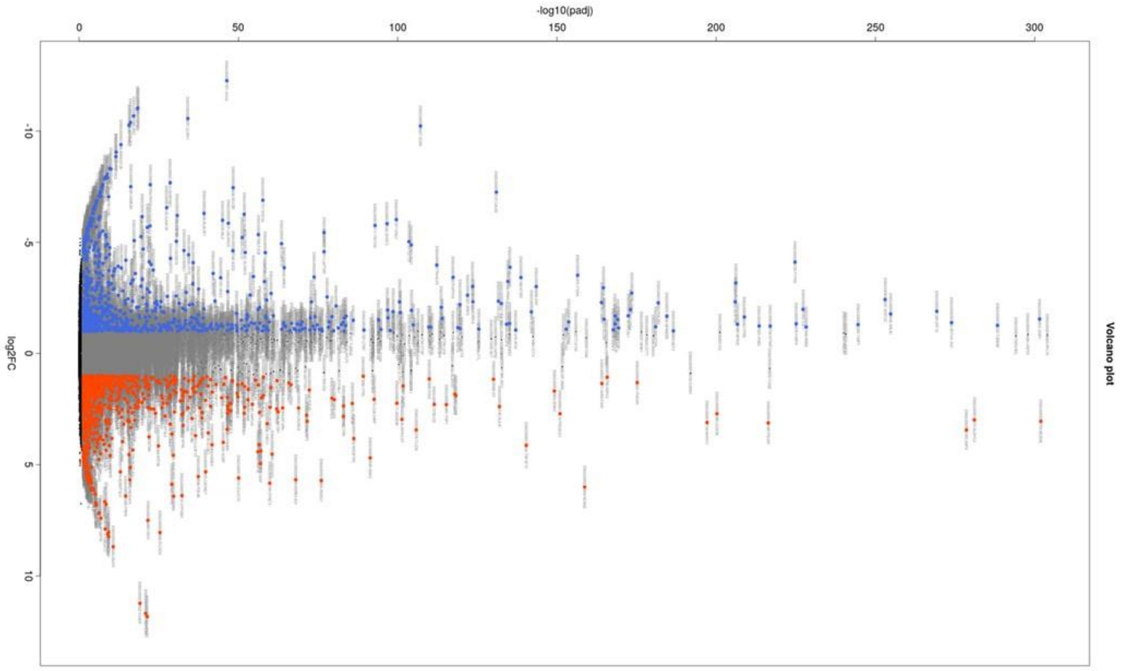

Fig. S3 M-A plot and volcano plot for MIA capa-2-GR vs -PR

Fig. S3. GR vs PR

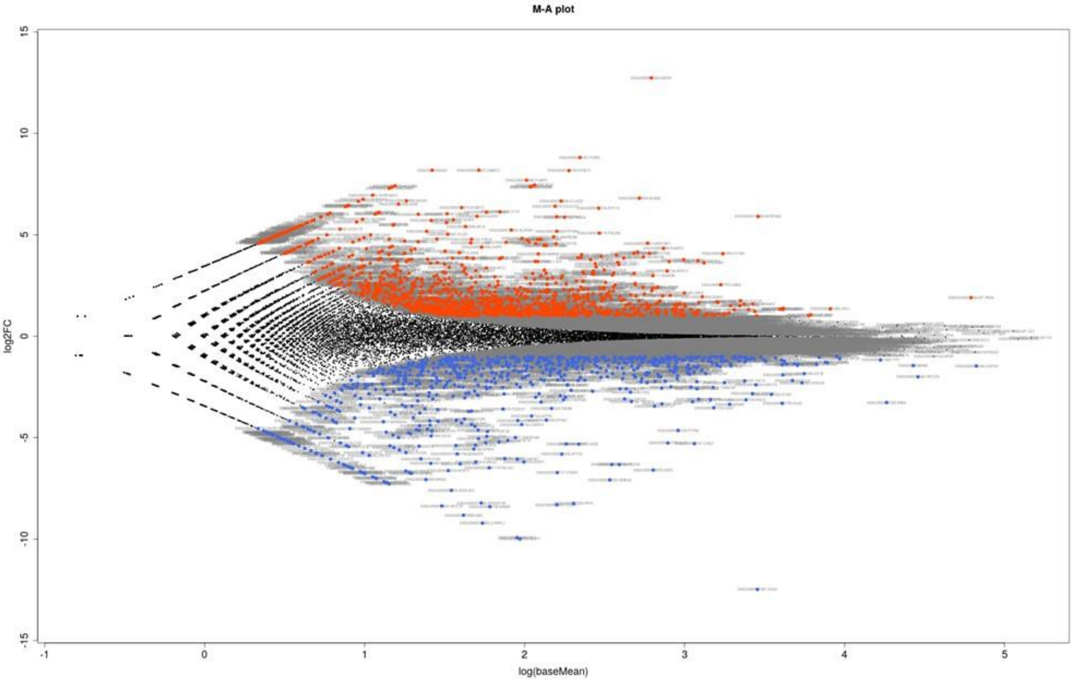

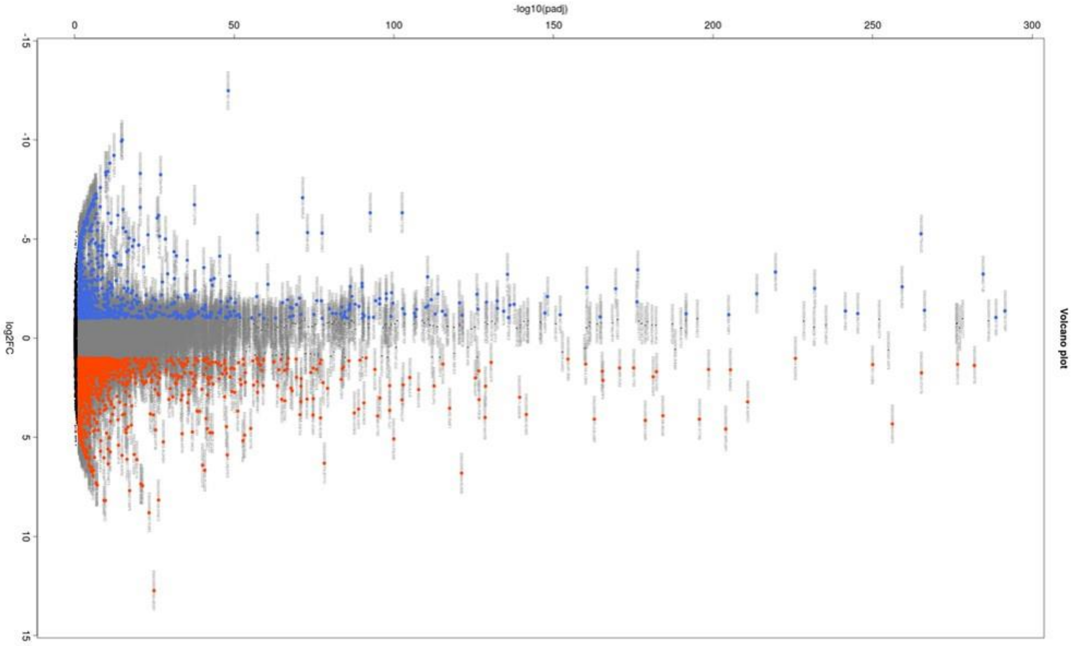

Table S1 primers for qPCR

Table S1. List of primers for qPCR.

STAT3	Forward	CTTTGAGACCGAGGTGTATCACC
	Reverse	GGTCAGCATGTTGTACCACAGG
GLI1	Forward	AGCCTTCAGCAATGCCAGTGAC
	Reverse	GTCAGGACCATGCACTGTCTTG
ZNF367	Forward	GGACAGCTCAAAACACATCAGCG
	Reverse	TTCGGACAGTGGCGGTTTGCAT
NKX3-2	Forward	CCGCTTCCAAAGACCTAGAGGA
	Reverse	ACCGTCGTCCTCGGTCCTTGG
ZIC2	Forward	ACACAGGCGAGAAACCCTTCCC
	Reverse	ACTCACACTGGAACGGCTTCTC
IFIT2	Forward	GGAGCAGATTCTGAGGCTTTGC
	Reverse	GGATGAGGCTTCCAGACTCCAA
HEY1	Forward	TGTCTGAGCTGAGAAGGCTGGT
	Reverse	TTCAGGTGATCCACGGTCATCTG
TAP1	Forward	GCAGTCAACTCCTGGACCACTA
	Reverse	CAAGGTTCCCACTGCTTACAGC
FBXL14	Forward	TGTACGGCTGCACCCGAATCAC
	Reverse	CTTCCCCGAGTTCTCACAGTGA
IQSEC3	Forward	CAGCAAGAAGCAGTTCAACCGC
	Reverse	TCAATGAGCCGCTCCACCTTCT
GASK1B	Forward	CATGAGTGGTCCAAGATGGCAC
	Reverse	ACACAGGCATCTTCCTTGCGAG
SCN1A	Forward	GGACTGTATGGAGGTTGCTGGT
	Reverse	GCAAGGTTGTCTGCACTAAATGAG
